# Emergence of *fosA3* and *bla*_*CTX–M–*__14_ in Multidrug-Resistant *Citrobacter freundii* Isolates From Flowers and the Retail Environment in China

**DOI:** 10.3389/fmicb.2021.586504

**Published:** 2021-02-05

**Authors:** Ke Cheng, Liang-Xing Fang, Qian-Wen Ge, Dong Wang, Bing He, Jia-Qi Lu, Zi-Xing Zhong, Xi-Ran Wang, Yang Yu, Xin-Lei Lian, Xiao-Ping Liao, Jian Sun, Ya-Hong Liu

**Affiliations:** ^1^National Risk Assessment Laboratory for Antimicrobial Resistance of Animal Original Bacteria, South China Agricultural University, Guangzhou, China; ^2^Guangdong Provincial Key Laboratory of Veterinary Pharmaceutics Development and Safety Evaluation, South China Agricultural University, Guangzhou, China; ^3^Guangdong Laboratory for Lingnan Modern Agriculture, Guangzhou, China; ^4^Jiangsu Co-Innovation Center for the Prevention and Control of Important Animal Infectious Disease and Zoonoses, Yangzhou University, Yangzhou, China

**Keywords:** flower, fosfomycin-resistance, *Citrobacter freundii*, *fosA3*, *bla*_*CTX–M–*__14_

## Abstract

We examined the prevalence and transmission of the *fosA3* gene among *Citrobacter freundii* isolates from flowers and the retail environments. We identified 11 fosfomycin-resistant *C. freundii* strains (>256 μg/mL) from 270 samples that included petals (*n* = 7), leaves (*n* = 2), dust (*n* = 1) and water (*n* = 1). These 11 isolates were multidrug-resistant and most were simultaneously resistant to fosfomycin, cefotaxime, ciprofloxacin and amikacin. Consistently, all 11 isolates also possessed *bla*_*CTX–M–*__14_, *bla*_*CMY–*__65__/__122_, *aac(6’)-Ib-cr*, *qnrS1*, *qnrB13/6/38* and *rmtB*. These *fosA3*-positive isolates were assigned to two distinct PFGE patterns and one (*n* = 9) predominated indicating clonal expansion of *fosA3*-positive isolates across flower markets and shops. Correspondingly, *fosA3* was co-transferred with *bla*_*CTX–M–*__14_ *via* two plasmid types by conjugation possessing sizes of 110 kb (*n* = 9) and 260 kb (*n* = 2). Two representatives were fully sequenced and p12-1 and pS39-1 possessed one and two unclassified replicons, respectively. These plasmids shared a distinctive and conserved backbone in common with *fosA3*-carrying *C. freundii* and other *Enterobacteriaceae* from human and food animals. However, the *fosA3*-*bla*_*CTX–M–*__14_-containing multidrug resistance regions on these untypable plasmids were highly heterogeneous. To the best of our knowledge, this is the first report of *fosA3* and *bla*_*CTX–M–*__14_ that were present in bacterial contaminants from flower shops and markets. These findings underscore a public health threat posed by untypable and transferable p12-1-like and pS39-1-like plasmids bearing *fosA3*-*bla*_*CTX–M–*__14_ that could circulate among *Enterobacteriaceae* species and in particular *C. freundi* in environmental isolates.

## Introduction

There are two major problems in the treatment of bacterial infections: the spread of multidrug-resistant (MDR) or extensively drug-resistant (XDR) pathogens and lack of development of new antibiotics active against these bacteria (Falagas et al., 2016). This situation has renewed interest in older antibiotics such as fosfomycin as alternatives or “last resort” therapies (Falagas et al., 2016; Sastry and Doi, 2016). Fosfomycin is viewed as a suitable empirical drug that has retained activity against resistant strains (Popovic et al., 2010). The World Health Organization has reclassified fosfomycin as a “critically important antimicrobial” based on its broad-spectrum bactericidal reactivity and good pharmacological properties (World Health Organization [WHO], 2011).

Clinical cases of fosfomycin resistance have increased in the last decade especially due to inactivation of the drug *via* plasmid-mediated fosfomycin-modification (*fos*) genes in *Enterobacteriaceae* (Diez-Aguilar et al., 2013). Fosfomycin covalently binds to a cysteine thiol in the active site of MurA and interferes with peptidoglycan synthesis at an earlier step than the action of β-lactams or glycopeptides. FosA is a glutathione S-transferase that covalently modifies fosfomycin for inactivation. There are currently more than 10 *fos* types, and *fosA*, its subtypes, and *fosC2* are primarily found in the *Enterobacteriaceae* (Yang et al., 2017). In China, plasmid-encoded *fosA3* in *Escherichia coli* isolates from food and pets have been reported with high detection rates (Hou et al., 2012; Yang et al., 2014; Yao et al., 2016; Wang et al., 2017) although fosfomycin has not been approved for veterinary use. Interestingly, *fosA3* is often found co-localized with *bla*_*CTX–M*_ on epidemic plasmids and this most likely promotes the transfer and dissemination of *fosA3* in humans and animals (Yang et al., 2014; Feng et al., 2015). Co-spread of *fosA3* with other important antibiotic resistance genes (ARG) is concerning due to the potential to rapidly develop into MDR *Enterobacteriaceae* strains.

Additionally, *E. coli* strains carrying *fosA3* and *bla*_*CTX–M–*__14_ have been isolated from vegetables in Netherlands (Freitag et al., 2018), even more *mcr-1* gene in *E. coli* was identified in fresh vegetables in Guangzhou, China (Luo et al., 2017). Literature also have claimed that plants could be contaminated by manure and wastewater from animal farming, contributing to the widely spread of AMR (Zeng et al., 2019; Sun et al., 2020). As a kind of plant, flowers are closely related to human life and possible vectors for AMR genes transformation. However, few reports have focused on the significance of flowers as a pathway for AMR spread.

Therefore, we investigated drug-resistant bacteria/drug-resistant genes from flowers and retail environment including water and dust in florists in Guangzhou, China. There are relatively few reports of plasmid-borne *fosA3* in *Citrobacter. freundii*, a bacterium associated with opportunistic nosocomial infections of the respiratory and urinary tracts and blood (Feng et al., 2015; Li M. et al., 2018). Herein, we present the first report of the emergence of *fosA3* in *C. freundii* isolates from flowers and the retail environments. We further investigated the molecular epidemiology of *fosA3*-carrying *C. freundii* isolates and characterized the *fosA3*-bearing plasmids.

## Materials and Methods

### Bacterial Strains and Detection of fos Genes Materials and Methods

A total of 270 samples were randomly collected from 3 flower markets and 6 flower shops in Guangzhou, China during March 2017. The samples included lily petals (*n* = 90), lily leaves (*n* = 90), dust (*n* = 45), and water (*n* = 45). Flowers and leaves were separately collected in sterile sealed bags. Water for watering flowers was collected in 50 mL centrifuge tubes. Dust samples were wiped with sterile cotton swabs in the surface dust from tables or floors (each 10-cm × 10-cm area) in retail shops, and then were rinsed in 2 mL sterile physiological saline solution. One petal and one leaf of each lily flowers were picked and washed with 10 mL of sterile saline solution. Then 100 μL of dust-resuspension, flower washed fluid and water samples were incubated in 4 mL drug-free LB broth for 12–16 h at 37°C and plated on MacConkey agar plates containing 256 μg/mL fosfomycin plus 25 μg/mL glucose-6-phosphate. After 18 h incubation at 37°C, 1–2 red colonies of different morphologies from each plate were selected. The bacterial species identification was performed using the MALDI-TOF MS (Shimadzu-Biotech, Japan) and 16S rRNA gene sequencing (16srRNA-F: AGAGTTTGATCATGGCTC; 16srRNA-R: GGTTACCTTGTTACGACTT). Fosfomycin-resistant *C. freundii* isolates (≥256 mg/L) were screened for the presence of the plasmid-mediated fosfomycin resistance genes *fosA1*, *fosA2*, *fosA3*, *fosA4*, *fosA5*, *fosA6*, *fosA7*, and *fosC2* by PCR amplification and sequencing using primers as previously described (Huang et al., 2020).

### Antimicrobial Susceptibility Testing and Resistance Genes Detecting

All *fos*-positive isolates were screened for the minimum inhibitory concentration (MIC) using the Mueller Hinton (MH) agar dilution method (MH agar was purchased from Huankai Co., Ltd., Guangzhou, China) and the results were interpreted according to CLSI (Clinical and Laboratory Standards Institute [CLSI], 2018) and veterinary CLSI (Clinical and Laboratory Standards Institute [CLSI], 2019) guidelines. The following antimicrobials were tested: fosfomycin (FOS), cefotaxime (CTX), amoxicillin-clavulanate (AMC), meropenem (MEM), tetracycline (TET), chlortetracycline (CTET), doxycycline (DOX), ciprofloxacin (CIP), gentamicin (GEN), amikacin (AMK), sulfamethoxazole/trimethoprim (SXT), florfenicol (FFC), chloramphenicol (CHL), and rifampicin (RIF) (above drugs were purchased from Sigma Chemical Co., St Louis, MO, United States). MICs of tigecycline and colistin were determined by MH broth microdilution (MH broth was purchased from Huankai Co., Ltd., Guangzhou, China) and the resistance breakpoint was interpreted according to EUCAST criteria (>2 μg/mL) and FDA criteria (≥8 μg/mL). *E. coli* ATCC 25922 was used as a quality control strain.

All *fos*-positive isolates were further screened for the presence of the extended spectrum β-lactamase (ESBL) gene *bla*_*CTX–M*__–__9__*G/*__1__*G*_, the plasmid-mediated AmpC β-lactamase gene *bla*_*CMY*_, the plasmid-mediated quinolone resistance (PMQR) genes *qnrA*, *qnrB*, *qnrS*, *aac-(6′)-Ib-cr*, *qepA*, *oqxA* and *oqxB*), *floR* as well as 16S-RMTase genes (*armA*/*rmtB*) using PCR amplification as previously described (Phuc Nguyen et al., 2009; Fang et al., 2016; Liu et al., 2016). Notably, *bla*_*CTX–M–*__9__*G*_-positive isolates were further tested by PCR amplification using previous primers of ESBL-encoding genes (IS*Ecp1*-F: CTATCCGTACAAGGGAGTGT; IS*903*-R: TTTCCACTCGCCTTCACC) and protocols to confirm the subtypes of ESBL-encoding genes and other genotypes. All PCR products were sent for sequencing and the DNA sequences were blasted with GenBank database^1^.

### Molecular Typing

Chromosomal DNA digested with *Xba*I restriction enzyme was used for PFGE (Gautom, 1997) to analyze the genetic relatedness of all isolates containing *fos*. PFGE patterns were analyzed with the Dice coefficient and the unweighted pair group method with average linkages (UPGMA) clustering method using BioNumerics software (Applied Maths, Sint-Martens-Latem, Belgium). PFGE types were defined with >90% similarity between clusters.

### Transfer of fos Genes, Gene Location and Plasmid Replicon Typing

To determine the transferability of *fosA3* genes, isolates positive for *fosA3* were selected for conjugation experiments using the broth-mating method and streptomycin-resistant *E. coli* strain C600 (MIC > 2000 μg/mL) as the recipient. The donor and recipient strains were inoculated into 4 mL LB broth (Huankai Co., Ltd., Guangzhou, China) and shaken at 37°C for 4 h, then the donors and recipients were mixed in a 1:4 (100 and 400 μL) ratio in a 2 mL EP tubes and incubated at 37°C for 20 h. Transconjugants were selected on MacConkey agar plates supplemented with 2000 μg/mL streptomycin and 256 μg/mL fosfomycin (Yang et al., 2014). Antimicrobial susceptibility testing of the transconjugants and co-transfer of other resistance genes were determined as mentioned above. Incompatibility (Inc) groups were determined using PCR-based replicon typing (PBRT) (Carattoli et al., 2005).

The bacterial cell of the transconjugants were lysed with the ESP buffer (0.5 M EDTA, pH 9.0; 1% sodium lauroyl sarcosinate; 1 mg of proteinase K per mL) and then the bacterial DNA was embedded in the gel block. The S1-PFGE protocol is detailed in previous reports (Barton et al., 1995). The *fosA3* gene genomic locations were identified by linearization of plasmids from transconjugants using S1 nuclease followed by PFGE (Yang et al., 2014). Southern blotting was carried out from S1-PFGE gels using a digoxigenin-labelled probe specific for *fosA3*.

### WGS Sequencing and Characterization of fosA3-Bearing Plasmids

Based on the results of plasmid analysis and PFGE typing, the total genomic DNA was extracted from two *C. freundii* strains S39 and H12-3-2 using a TIANamp Bacteria DNA Kit (Tiangen) and DNA libraries were constructed with 250-bp paired-end whole-genome sequencing using the Illumina HiSeq system (Illumina, San Diego, CA, United States) (Ma et al., 2020). The obtained paired-end Illumina reads were assembled *de novo* using SPAdes v3.6.2 (default parameters except –careful and –k 21,33,55,77,99,127) (Bankevich et al., 2012). In addition, to obtain long reads sequence, selected strains were further sequenced using Oxford Nanopore MinION flowcell R9.4 (Li R. et al., 2018). *De novo* hybrid assembly was performed using a combined Illumina HiSeq and Nanopore sequencing approach (Nextomics). Genome assembly was performed with Unicycler version 0.4.1 (Wick et al., 2017) using a combination of short and long reads, followed by error correction with Pilon version 1.12 (Walker et al., 2014). Gene prediction and annotation were performed using RAST (Overbeek et al., 2014)^2^ and BLAST^3^ and rechecked manually. Alignments with highly homologous sequences (with >90% coverage and >90% nucleotide identity) from the NCBI database and generation of plasmid maps were performed with BRIG (Alikhan et al., 2011) and Easyfig (Sullivan et al., 2011).

### Nucleotide Sequence Accession Numbers

The representative *fosA3*-bearing genome sequences S39 and H12-1-2 were submitted to NCBI with the accession numbers CP045555 and CP045837, respectively, and *fosA3*-bearing plasmids sequences pS39-1 and pH12-1 with the accession numbers CP045556 and CP045838, respectively.

## Results

### Antimicrobial Susceptibility and Prevalence of fosA3 Genes

We isolated 17 fosfomycin-resistant Enterobacteriaceae from flower retail environments including 11 *C. freundii* and 6 *E. coli* from our group of 270 samples. The 11 *C. freundii* isolates were from 2 flowers markets and 3 flowers shops and samples included petals (*n* = 7), leaves (*n* = 2), dust (*n* = 1), and water (*n* = 1). All 11 isolates possessed the *fosA3* gene and no other *fos* gene was detected by PCR. These 11 were all were concurrently resistant to fosfomycin, cefotaxim, amoxicillin-clavulanate, tetracycline, chlortetracycline, doxycycline, ciprofloxacin, gentamicin, sulfamethoxazole/trimethoprim, florfenicol, chloramphenicol, and rifampin (Table 1). In addition, most of these isolates were also resistant to amikacin with MICs > 256 μg/mL (*n* = 9). However, all *fosA3*-positive isolates were susceptible to meropenem and tigecycline.

**TABLE 1:** Background information and characteristics of *fosA3*-positive *Citrobacter freundii*.

**Strains^1^**	**Locations**	**Source**	**PFGE Typing**	**Resistance genes^2^**	**Resistance profiles^3^**	**Replicon type^4^**	**Plasmids size (kb)**
H12-3-2	Flower market 1	Lily petals	I	*fosA3, bla*_*CTX–M–*__14_, *bla*_*CMY–*__122_, *floR, aac(6’)-Ib-cr, rmtB, qnrB13, qnrS1*	FOS, CTX, AMC, TET, CETE, DOX, CIP, GEN, AMK, SXT, FFC, CHL, RFP	UT	∼110
H41-7-2	Flower market 2	Lily petals	I	*fosA3, bla*_*CTX–M–*__14_, *bla*_*CMY*_, *floR, aac(6’)-Ib-cr, rmtB, qnrB, qnrS*	FOS, CTX, AMC, TET, CETE, DOX, CIP, GEN, AMK, SXT, FFC, CHL, RFP	UT	∼110
HR5	Flower market1	Dust	I	*fosA3, bla*_*CTX–M–*__14_, *bla*_*CMY*_, *floR, aac(6’)-Ib-cr, rmtB, qnrB, qnrS*	FOS, CTX, AMC, TET, CETE, DOX, CIP, GEN, AMK, SXT, FFC, CHL, RFP	UT	∼110
N21-1	Flower shop 1	Lily petals	I	*fosA3, bla*_*CTX–M–*__14_, *bla*_*CMY*_, *floR, aac(6’)-Ib-cr, rmtB, qnrB, qnrS*	FOS, CTX, AMC, TET, CETE, DOX, CIP, GEN, AMK, SXT, FFC, CHL, RFP	UT	∼110
N52-2	Flower shop 2	Lily petals	I	*fosA3, bla*_*CTX–M–*__14_, *bla*_*CMY*_, *floR, aac(6’)-Ib-cr, rmtB, qnrB, qnrS*	FOS, CTX, AMC, TET, CETE, DOX, CIP, GEN, AMK, SXT, FFC, CHL, RFP	UT	∼110
N89-1-1	Flower shop 3	Lily petals	I	*fosA3, bla*_*CTX–M–*__14_, *bla*_*CMY*_, *floR, aac(6’)-Ib-cr, rmtB, qnrB, qnrS*	FOS, CTX, AMC, TET, CETE, DOX, CIP, GEN, AMK, SXT, FFC, CHL, RFP	UT	∼110
NW2-2	Flower shop 1	Water	I	*fosA3, bla*_*CTX–M–*__14_, *bla*_*CMY*_, *floR, aac(6’)-Ib-cr, rmtB, qnrB, qnrS*	FOS, CTX, AMC, TET, CETE, DOX, CIP, GEN, AMK, SXT, FFC, CHL, RFP	UT	∼110
S66-1-1	Flower shop 3	Lily petals	I	*fosA3, bla*_*CTX–M–*__14_, *bla*_*CMY*_, *floR, aac(6’)-Ib-cr, rmtB, qnrB, qnrS*	FOS, CTX, AMC, TET, CETE, DOX, CIP, GEN, AMK, SXT, FFC, CHL, RFP	UT	∼110
S40-1-2	Flower shop 2	Lily petals	I	*fosA3, bla*_*CTX–M–*__14_, *bla*_*CMY*_, *floR, aac(6’)-Ib-cr, rmtB, qnrB, qnrS*	FOS, CTX, AMC, TET, CETE, DOX, CIP, GEN, AMK, SXT, FFC, CHL, RFP	UT	∼110
S39	Flower shop 2	Lily leaf	II	*fosA3, bla*_*CTX–M–*__14_, *bla*_*CMY*_, *floR, aac(6’)-Ib-cr, qnrB38, qnrB6, qnrS1*	FOS, CTX, AMC, TET, CETE, DOX, CIP, GEN, SXT, FFC, CHL, RFP	UT	∼261
S45-1	Flower shop 3	Lily leaf	II	*fosA3, bla*_*CTX–M–*__14_, *bla*_*CMY*_, *floR, aac(6’)-Ib-cr, qnrB, qnrB, qnrS*	FOS, CTX, AMC, TET, CETE, DOX, CIP, GEN, SXT, FFC, CHL, RFP	UT	∼261

*^1^WGS sequence are underlined. ^2^Resistance phenotypes transferred to the recipient by conjugation are underlined. FOS, fosfomycin; CTX, cefotaxime; AMC, amoxicillin-clavulanate; MEM, meropenem; TIG, tigecycline; TET, tetracycline; CTET, chlortetracycline; DOX, doxycycline; CIP, ciprofloxacin; GEN, gentamicin; AMK, amikacin; SXT, sulfamethoxazole/trimethoprim; FFC, florfenicol; CHL, chloramphenicol; RIF, rifampicin. ^3^Transfer of *fosA3* and co-transfer of this gene with other genotypes and phenotypes are underlined. ^4^UT, Untypeable.*

We further screened these 11 *fosA3*-positive *C. freundii* isolates for the presence of other important ARGs and identified numerous gene combinations. These included *bla*_*CTX–M–*__14_*-bla*_*CMY*_*-rmtB-aac(6’)-Ib-cr-qnrB-qnrS-floR* (*n* = 9) and *bla*_*CTX–M–*__14_*-bla*_*CMY*_*-aac(6’)-Ib-cr-qnrB-qnrB-qnrS-floR* (*n* = 2) (Table 1).

### Pulsed-Field Gel Electrophoresis (PFGE) Typing

**FIGURE 1:**
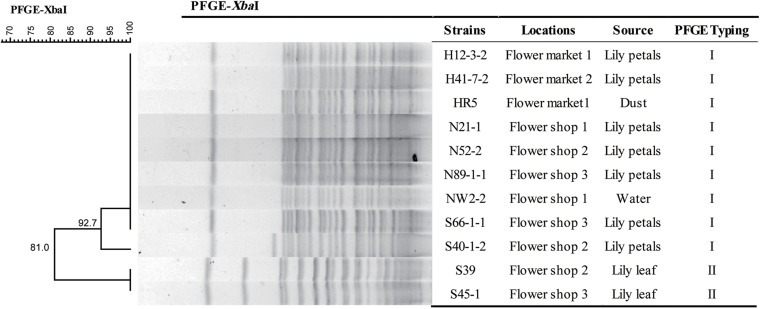
PFGE dendrogram showing the genetic relatedness of 13 *Citrobacter freundii* isolates including 2 *fosA3*-positive isolates.

We successfully performed PFGE typing for all 11 *fosA3-*positive *C. freundii* isolates. We found two different PFGE patterns that retained >90% similarity and were designated types I and II. Type I predominated and contained nine *C. freundii* isolates recovered from Lily petals, dust and water from two markets and three flower shops (Figure 1).

### Location of *fosA3* Gene and Transferability of Plasmids Carrying *fosA3*

All 11 *fosA3*-positive isolates were able to mobilize and transfer *fosA3* to recipient strain *E. coli* C600. These transconjugants were highly resistant to FOS (MIC > 512 μg/mL) with high-level resistance to CTX (MICs ≥ 64 μg/mL). In addition, two transconjugants also showed high-level resistance to TET, CETE, GEN, SXT, CHL, and RFP (MICs ≥ 64 μg/mL) and their MICs to DOX and CIP were increased 16- and 32-fold, respectively, when compared with the recipient strain (Table 1 and Supplementary Table S1). Correspondingly, *bla*_*CTX–M–*__14_ (*n* = 11), *aac(6’)-Ib-cr* (2) and *qnrB* (*n* = 2) were co-transferred with *fosA3* in 11 donors (Table 1). Gene location and plasmid replicon typing demonstrated that the transferable *fosA3* gene coexisted with *bla*_*CTX–M–*__14_ (*n* = 9) and *bla*_*CTX–M–*__14_-*aac(6’)-Ib-cr*-*qnrS* (*n* = 2) that were present on the untypable plasmids with sizes of 110 kb (*n* = 9) and 260 kb (*n* = 2), respectively (Table 1 and Supplementary Figure S1).

### *fosA3*-Carrying Plasmid Analysis

Based on the results of PFGE typing and plasmid analysis, we selected two representative *C. freundii* strains S39 and H12-3-2 for complete sequencing. Strain S39 contained one chromosome (4,806,164 bp) and 4 plasmids including one *fosA3*-carrying plasmid pS39-1. Strain H12-3-2 contained one chromosome (4,948,530 bp) and harbored 2 plasmids including a *fosA3*-carrying plasmid pH12-1. BWA (Li and Durbin, 2009) and Samtools (Li et al., 2009) software were used to obtain the sequence depth of each base, and the average sequencing depths of each nucleotide were calculated. The chromosome sequence of strain H12-1-2 was covered 96 × on average, and the chromosome sequence of strain S39 was covered 159 × on average. The plasmid sequence of pH12-1-1 was covered 106 × on average, and the plasmid sequence of pS39-1 was covered 90 × on average. In addition to the presence of *fosA3*, *bla*_*CTX–M–*__14_, *bla*_*CMY–*__65_, *aac(6’)-Ib-cr, qnrB6, qnrS1* and *qnrB38*, strain S39 also harbored *bla*_*TEM–*__1__*B*_, *catA2, floR, aac(3)-IIa, aadA16, aph(3”)-Ib, aph(6)-Id, sul1, sul2, dfrA27, tet(A), tet(D)*, and *arr-3*. In addition to *fosA3, bla*_*CTX–M–*__14_, *bla*_*CMY–*__122_, *aac(6’)-Ib-cr, qnrS1, qnrB13*, and *rmtB*, strain H12-3-2 also harbored *bla*_*OXA–*__1_, *bla*_*TEM–*__1__*B*_, *catB3, floR, aadA2b, aph(3”)-Ib, aph(6)-Id, sul1, sul2, dfrA12*, and *arr-3*. These ARGs were all located on plasmids except for the chromosomal *qnrB38* and *bla*_*CMY–*__65_ in strain S39 and chromosomal *qnrB13 and bla*_*CMY–*__122_ in strain H12-3-2. Furthermore, ARG carriage was consistent with the resistance phenotypes for these strains (Table 1).

**FIGURE 2:**
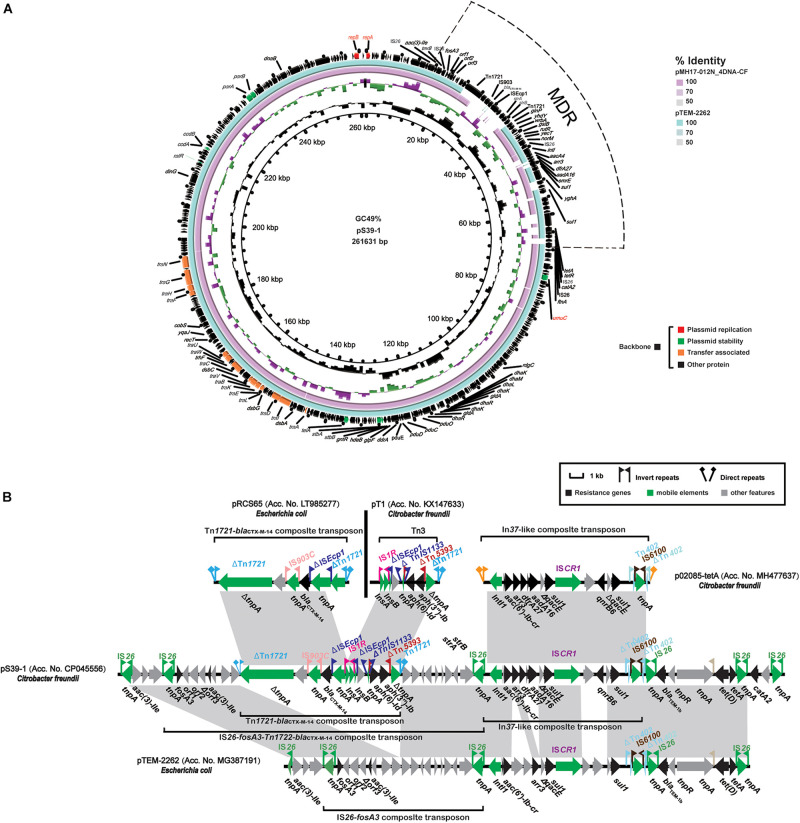
Characteristics of the complete nucleotide sequences of the *fosA3* plasmid pS39-1 identified in this study. **(A)** A comparison of pS39-1 with two untypable *fosA3* plasmids pTEM-2262 (Acc. No. MG387191) and pMH17-012N_4DNA-CF (Acc. No. AP018570). **(B)** Comparison of the *fosA3*-containing MRR with the corresponding regions from pRCS65 (Acc. No. LT985277), pT1 (Acc. No. KX147633), p02085-tetA (Acc. No. MH477637), and pTEM-2262 (Acc. No. MG387191). Genes are denoted by arrows. Genes, mobile elements, and other features are colored based on functional classification. Shaded regions denote shared DNA homology (>95% nucleotide identity).

Plasmid pS39-1 was 261, 631 bp in length and included 332 coding sequences (CDS), an overall GC content of 49% and was untypable. The backbone sequence of pS39-1 was almost identical to the *fosA3*-bearing plasmids pMH17-012N_4 (Acc. No. AP018570) and pTEM-2262 (Acc. No. MG387191) (Figure 2A). These three plasmids contained 203 kb conserved backbone regions and the primary differences contained between *repA* (plasmid replication) and *umuC* (plasmid stability) that included large multidrug-resistance region (MRR). The conserved backbone regions encoding functions for plasmid replication (*repA* and *repB*), stability (*parAB*, *stbAB*, *ccdAB*, *ralR*, *telA*, *umuC*, and *higA*) and transfer (15 *tra/trh* genes). Of note, *repA* (837 bp) and *repB* (1119 bp) located on plasmid pS39-1 belonged to unclassified replicons and were nearly identical to the corresponding *repA* and *repB* genes of plasmids pMH17-012N_4 and pTEM-2262 with 0 and 1 SNP differences for *repA* and *repB*, respectively.

In addition to the plasmid backbone region, plasmid pS39-1 possessed a 53.4 kb MDR containing 15 ARGs (*fosA3*, *bla*_*CTX–M–*__14_, *bla*_*TEM–*__1__*B*_, *catA2*, *aac(3)-IIa*, *aadA16*, *aac(6’)-Ib-cr*, *aph(3”)-Ib, aph(6)-Id*, *sul1*, *qnrB6*, *dfrA27*, *tet(A)*, *tet(D)*, and *arr-3*) interspersed with different complete or truncated insertion sequences and transposons (IS*26*, ΔTn*1721*, IS*903C*, ΔIS*Ecp1*, IS*1R*, IS*CR1*, ΔTn*402*, and IS*6100*) (Figure 2A). This 53.4 kb MDR was highly similar to that in *fosA3*-bearing plasmid pTEM-2262. The primary difference between the two were full and partial deletions of the Tn*1721*-*bla*_*CTX–M–*__14_-composite transposon (13,196 bp) and the In*37-like* composite transposon (12,959 bp), respectively. Of note, the Tn*1721*-*bla*_*CTX–M–*__14_-composite transposon on pS39-1 was composed of two parts that were almost identical to the Tn*1721*-*bla*_*CTX–M–*__14_-composite transposon (10,459 bp) on pRCS65 (Acc. No. LT985277) and the Tn*3* transposon (4569 bp) on pT1 (Acc. No. KX147633), respectively. Furthermore, the In*37-like* composite transposon of plasmid pS39-1 was highly similar to that in plasmid p02085-tetA (Acc. No. MH477637) (Figure 2B).

**FIGURE 3:**
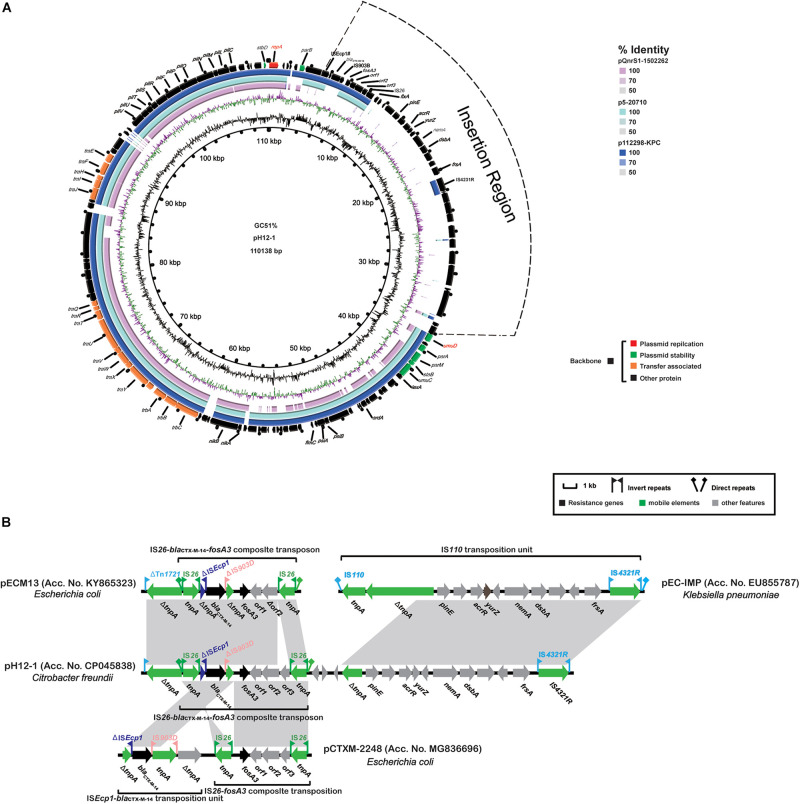
Characteristics of the complete nucleotide sequences of *fosA3*-carrying pH12-1 identified in this study. **(A)** A comparison with two *fosA3*-carrying untypeable plasmids p112298-KPC (Acc. No. KP987215), pQnrS1-1502262 (Acc. No. CP031572), and p5-20710 (Acc. No. CP030079). **(B)** Comparison of the *fosA3*-containing MRR in pH12-1 with the corresponding regions of pECM13 (Acc. No. KY865323), pEC-IMP (Acc. No. EU855787), and pCTXM-2248 (Acc. No. MG836696). See Figure 2 for annotation features.

Plasmid pH12-1 was 110, 138 bp in length and contained 117 CDSs and an overall GC content of 51%. The plasmid was untypable although its replication gene *repA*(867 bp) was similar to orthologs of 261 bp on the IncFII plasmids pAMA1167-NDM-5 (Acc. No. CP024805) and pRSB107 (Acc. No. AJ851089) with a 69.7% coverage and 73.6% identity. Sequence comparisons demonstrated that the backbone region of plasmid p12-1 was highly similar to corresponding regions on plasmids pQnrS1-1502262 (Acc. No. CP031572) and p5-20710 (Acc. No. CP030079) and p112298-KPC (Acc. No. KP987215) except that pH12-1 possessed a 32.7 kb insertion between the *parB* (plasmid partition) and *umuD* (DNA polymerase V subunit) genes (Figure 3A). The conserved backbone regions in plasmid pH12-1 (91.4 kb) contained *repA*, *parABM*, *umuCD*, *stbD*, *staB*, and 16 *tra*/*trh* transfer gene modules. The 32.7 kb insertion region was primarily composed of hypothetical proteins except for the structure Δ*tnpA*-*pinE*-*acrR*-*yurZ*-*dsbA*-*frsA*-IS*4321R* (11.3 kb) and the resistance region containing *bla*_*CTX–M–*__14_ and *fosA3* (7.1 kb). The former was highly similar to that in pEC-IMP (Acc. No. EU855787) and the latter was highly similar to *fosA3*-*bla*_*CTX–M–*__14_-carrying plasmids pECM13 (Acc. No. KY865323) and pCTXM-2248 (Acc. No. MG836696) except for partial gene deletions (Figure 3B).

## Discussion

Fosfomycin plays a critical role against MDR and XDR Gram-negative pathogens and in particular, it commonly plays a synergistic role paired with β-lactams and aminoglycosides in treating urinary tract infections (Sastry and Doi, 2016). However, the occurrence and dissemination of plasmid-borne fosfomycin resistance genes, especially *fosA3*, has cast a shadow over the use of fosfomycin in clinics. In this study, we identified *fosA3*-carrying *C. freundi* isolates with a detection rate of 4.1% among 270 samples from flowers and the retail environments in Guangzhou, China. The prevalence of *fosA3* among *C. freundii* strains from flowers was relatively low when compared with *E. coli* isolated from food animals (8.8%, during 2009–2011; 10.5%, during 2015–2016) in China (Yang et al., 2014; Wang et al., 2017). However, our *fosA3* detection rate was consistent with the recent report that *fosA3* sporadically occurs in *C. freundii* and other *Enterobacteriaceae* including *Salmonella spp.*, *Proteus mirabilis*, and *Enterobacter fergusonii* from humans, food animals, pets, retail meat as well as wild birds (Lin and Chen, 2015; Villa et al., 2015; Wong et al., 2016; Yao et al., 2016; Fang et al., 2019). Perhaps of great concern is the emergence of the *fosA3* gene in different *Enterobacteriaceae* species from these diverse origins.

Plants including vegetables and flowers can be contaminated with ARGs *via* wastewater irrigation or manure application. Interestingly, an MDR *C. freundii* strain (WCHCF65) in sewage from a Chinese hospital carried multiple clinically significant ARGs including *bla*_*CTX–M–*__12_, *bla*_*CTX–M–*__14_, *bla*_*SHV–*__12_, *bla*_*NDM–*__1_ and *bla*_*KPC–*__2_ as well as *fosA3* (Wu et al., 2016). Additionally, *mcr-1* was present with *fosA3* and *bla*_*CTX–M–*__14_ in *Raoultella ornithinolytica* and *E. coli* isolates from retail vegetables in Guangzhou, China (Luo et al., 2017). MDR *E. coli* strains co-harboring *fosA3* and *bla*_*CTX–M–*__14_ also occurred in fresh vegetables in Netherlands (Freitag et al., 2018). These observations indicate that plants are potential ARG reservoirs including *fosA3*.

In the present study, we identified the presence of *fosA3* on flowers and the retail environments. These *fosA3*-positive *C. freundi* isolates exhibited resistance to most of the tested antibiotics including cefotaxime, ciprofloxacin, and amikacin in addition to fosfomycin. Consistently we found that *fosA3* coexisted with the ESBL *bla*_*CTX–M–*__14_, the pAmpCs *bla*_*CMY–*__65_ and *bla*_*CMY–*__122_, the PMQR genes *aac(6’)-Ib-cr*, *qnrS1*, *qnrB13/qnrB6/qnrB38* as well as *rmtB*. Additionally, WGS analysis demonstrated that *bla*_*CMY–*__65_*/bla*_*CMY–*__122_ and *qnrB13/qnrB38* were located in the chromosome. This was consistent with the origin of plasmid-mediated *qnrB* and *bla*_*CMY–*__2_ -like genes from the chromosome of *Citrobacter spp.* (Verdet et al., 2009; Jacoby et al., 2011; Liao et al., 2015). Importantly, flowers contaminated with MDR bacteria will most likely come into direct contact with humans complicating the treatment and management of disease.

In this study, the *fosA3*-carrying *C. freundii* isolates were genetically related as judged by their PFGE profiles. In particular, we found an epidemic PFGE type that was composed of isolates from diverse origins across flower markets and shops. This indicated possible clonal dissemination of MDR *fosA3*-positive *C. freundii* isolates from flower markets and shops in a local region.

The spread of bacterial plasmids is an increasing global problem contributing to widespread ARG dissemination (San Millan, 2018). Several plasmid types are associated with the spread of *fosA3* and in particular, the epidemic IncF33:A-:B- and ST3-IncHI2 plasmids in *Enterobacteriaceae* from pets and food animals (Hou et al., 2012; Yang et al., 2014; Fang et al., 2019). However, we found that all our 11 *fosA3*-carrying plasmids including p12-1 and pS39-1 in *C. freundi* could not be assigned to any known *Enterobacteriaceae* incompatibility group. The single replicon gene *repA* possessed in plasmid p12-1 was highly similar to that in plasmid p112298-KPC where the *repA* was assigned to the IncFII RepA superfamily (Feng et al., 2015). Plasmid p12-1 showed a similar backbone region to the untypable *fosA3*-bearing plasmids in *E. hormaechei* and *C. freundii* isolates from humans in China and the United States (Wang et al., 2018; Figure 3).

Interestingly, a similar scenario was also observed for the other untypable *fosA3*-bearing plasmid pS39-1. Linear genomic comparisons revealed that a conserved backbone, including the two unclassified replicons *repA* and *repB*, were identified between pS39-1 and another two untypable *fosA3*-bearing plasmids pTEM-2262 and pMH17-012N_4 in *C. freundii* isolates of pig and human origin from China (Li M. et al., 2018; Zhang et al., 2019; Figure 2). Noticeably, pTEM-2262 also shared a conserved backbone with another four untypable non-*fosA3*-bearing plasmids from different species including *C. freundii*, *Kosakonia radicincitans*, and *Citrobacter werkmanii* with origins including the environment, vegetables and humans (Zhou et al., 2017; Zurfluh et al., 2017; Becker et al., 2018; Barry et al., 2019). These indicated that the untypable and conjugal p12-1-like and pS39-1-like plasmids could act as vectors for *fosA3* transmission between different *Enterobacteriaceae* from different ecological niches.

In contrast to the conserved backbones, the *fosA3*-*bla*_*CTX–M–*__14_-containing MDR of these untypable plasmids from the GenBank were highly heterogeneous. This was primarily due to acquisition or deletion of resistance determinants mediated by mobile genetic elements and recombination. Plasmid pS39-1 possessed a large *fosA3*-containing MRR composed of 15 ARGs and diverse insertion sequences and transposons. Furthermore, the large *fosA3*-containing MRRs in plasmid pS39-1 partially resembled analogous regions from different plasmids indicating the MRR likely originated from the recombination of genetic contents from different plasmids as previously described (Hammerum et al., 2016; Li M. et al., 2018; Jing et al., 2019; Maherault et al., 2019). Unlike pS39-1, an additional insertion region mainly composed of hypothetical proteins was also integrated into the variable region of plasmid p12-1 in addition to the *fosA3*-*bla*_*CTX–M–*__14_-containing resistance region. Interestingly, heterogeneous *fosA3*-containing multidrug resistance regions have been also identified on the epidemic ST3-IncHI2 and F33:A-:B- plasmids with a conserved backbone in *Salmonella* (Yang et al., 2014). These data indicated that diverse and flexible transmission of *fosA3* was associated with heterogeneous MRRs and conserved backbones of a specific group of plasmids including ST3-IncHI2 and F33:A-:B- as well as untypable replicons in *Enterobacteriaceae*.

In conclusion, this study revealed the presence of *fosA3* that co-existed with *bla*_*CTX–M–*__14_ in MDR *C. freundii* isolates from flowers and the retail environments. Clonal expansion and horizontal transmission of untypable plasmids were involved in the spread of *fosA3* and *bla*_*CTX–M–*__14_ among these *C. freundi* isolates. To the best of our knowledge, this is the first report of the identification of *fosA3* in bacteria isolated from flower shops and markets. The *fosA3*- and *bla*_*CTX–M–*__14_-bearing untypable and transferable p12-1-like and pS39-1-like plasmids could circulate among diverse *Enterobacteriaceae* species from diverse origins, including plants and humans. Future studies are necessary to monitor the prevalence and transmission of these plasmids in *Enterobacteriaceae* species especially *C. freundi*, to better understand the potential threat to public health.

## Data Availability Statement

The datasets presented in this study can be found in online repositories. The names of the repository/repositories and accession number(s) can be found in the article/ Supplementary Material.

## Author Contributions

Y-HL, JS, and X-PL designed the study. KC, L-XF, DW, BH, Q-WG, J-QL, and Z-XZ performed the experiments and collected the data. KC, L-XF, DW, BH, Q-WG, J-QL, Z-XZ, X-LL, YY, and X-RW analyzed and interpreted the data. KC wrote the draft of the manuscript. L-XF, Y-HL, JS, X-PL, and X-LL edited and revised the manuscript. Y-HL and JS coordinated the whole project. All authors contributed to the article and approved the submitted version.

## Conflict of Interest

The authors declare that the research was conducted in the absence of any commercial or financial relationships that could be construed as a potential conflict of interest.

## Footnotes

1 http://www.ncbi.nlm.nih.gov/

2 https://rast.nmpdr.org/

3 https://blast.ncbi.nlm.nih.gov/Blast.cgi
