# Supplementary material for: Emergence of fosA3 and blaCTX–M–14 in Multidrug-Resistant Citrobacter freundii Isolates From Flowers and the Retail Environment in China
Source: Front Microbiol. 2021 Feb 5;12:586504. doi: 10.3389/fmicb.2021.586504 (PMC7893115; doi:10.3389/fmicb.2021.586504)
Supplement: Supplementary file 1 [file Data_Sheet_1.pdf]

Supplementary Information for

Title: Emergence of *fosA3* and *bla*$_{CTX-M-14}$ in multidrug-resistant *Citrobacter freundii* isolates from flowers and their surrounding environments in China

Ke Cheng[1†], Liang-Xing Fang[1,3†], Bing He[1], Qian-wen Ge[1], Jia-qi Lu[1], Zi-xing Zhong[1], Xi-ran Wang[1], Yang Yu[1,3], Xin-lei Lian[1,3], Xiao-Ping Liao[1,2,3], Jian Sun[1,2,3*], Ya-Hong Liu[1,2,3,4*]

[1]National Risk Assessment Laboratory for Antimicrobial Resistance of Animal Original Bacteria, South China Agricultural University, Guangzhou 510642, China

[2]Guangdong Laboratory for Lingnan Modern Agriculture, Guangzhou 510642, China

[3]Guangdong Provincial Key Laboratory of Veterinary Pharmaceutics Development and Safety Evaluation, South China Agricultural University, Guangzhou 510642, China

[4]Jiangsu Co-Innovation Center for the Prevention and Control of Important Animal Infectious Disease and Zoonosis, Yangzhou University, Yangzhou 225009, PR China

[†]Both authors contributed equally to this work
*****Corresponding Author
Jian Sun, PhD, Laboratory of Veterinary Pharmacology, College of Veterinary Medicine, South China Agricultural University, Guangzhou 510642, PR. China. E-mail: jiansun@scau.edu.cn. Tel: +86-020-85285507; Fax: +86-020-85285507

Ya-Hong Liu, PhD, Laboratory of Veterinary Pharmacology, College of Veterinary Medicine, South China Agricultural University, Guangzhou 510642, PR. China. E-mail: lyh@scau.edu.cn. Tel: +86-20-85280006; Fax: +86-20-85280006

Table S1. Antimicrobial susceptibility testing among 11 *fosA3*-positive *Citrobacter freundii* isolates and their corresponding transconjugants (MICs, µg/mL).

	FOS	CTX	AMC	MEM	TIG	TET	CTET	DOX	CIP	GEN	AMK	SXT	FFC	CHL	RIF	CS
H12-3-2	>512	128	64	0.016	1	128	128	32	32	128	>256	320	256	128	256	1
H12-3-2	>512	64	4	0.016	0.25	2	2	1	0.016	1	1	2.5	2	2	4	0.25
H41-7-2	>512	128	64	0.016	1	128	64	32	32	128	>256	320	256	128	256	1
H47-1-2	>512	64	4	0.016	0.25	2	2	1	0.016	1	1	2.5	2	2	4	0.25
HR5	>512	128	64	0.016	1	128	128	32	32	128	>256	320	256	128	256	1
HR5	>512	64	4	0.016	0.25	2	2	1	0.016	1	1	2.5	2	2	4	0.25
N21-1	>512	128	64	0.016	1	128	128	32	32	128	>256	320	256	128	256	1
N21-1	>512	64	4	0.016	0.25	2	2	1	0.016	1	1	2.5	2	2	4	0.25
N52-2	>512	128	64	0.016	1	128	128	32	32	128	>256	320	256	128	256	1
N52-2	>512	64	4	0.016	0.25	2	2	1	0.016	1	1	2.5	2	2	4	0.25
N89-1-1	>512	128	64	0.016	1	256	128	32	32	128	>256	320	256	128	256	1
N89-1-1	>512	64	4	0.016	0.25	2	2	1	0.016	1	1	2.5	2	2	4	0.25
NW2-2	>512	128	64	0.016	1	128	128	32	32	128	>256	320	256	128	256	1
NW2-2	>512	64	4	0.016	0.25	2	2	1	0.016	1	1	2.5	2	2	4	0.25
S66-1-1	>512	128	64	0.016	1	128	128	32	32	128	>256	320	256	128	256	1
S66-1-1	>512	32	4	0.016	0.25	2	2	1	0.016	1	1	2.5	2	2	4	0.25
S40-1-2	>512	128	64	0.016	1	128	128	32	32	128	>256	320	256	128	256	1
S40-1-2	>512	64	4	0.016	0.25	2	2	1	0.016	1	1	2.5	2	2	4	0.25
S39	>512	128	32	0.03	1	256	128	32	16	128	1	320	256	256	>256	1
S39	>512	64	4	0.016	0.25	64	128	16	0.5	128	1	320	2	64	64	0.25
S45-1	>512	128	32	0.03	1	256	128	32	16	128	1	320	256	256	>256	1
S45-1	>512	64	4	0.016	0.25	64	128	16	0.5	64	1	320	2	64	64	0.25
C600	4	0.03	4	0.016	0.25	1	2	1	0.016	2	1	5	1	2	2	0.25
25922	2	0.03	4	0.016	0.25	2	2	1	0.016	1	1	2.5	2	2	2	0.25

[1] FOS, fosfomycin; FFC, florfenicol; CTX, cefotaxime; AMC, amoxicillin-clavulanate; MEM, meropenem; TIG, tigecycline; TET, tetracycline; CTET, chlortetracycline; DOX, doxycycline; CIP, ciprofloxacin; GEN, gentamicin; AMK, amikacin; SXT, sulfamethoxazole/trimethoprim; CHL, chloramphenicol; RIF, rifampicin

[2] Transconjugants that were co-transferred by conjugation are underlined.

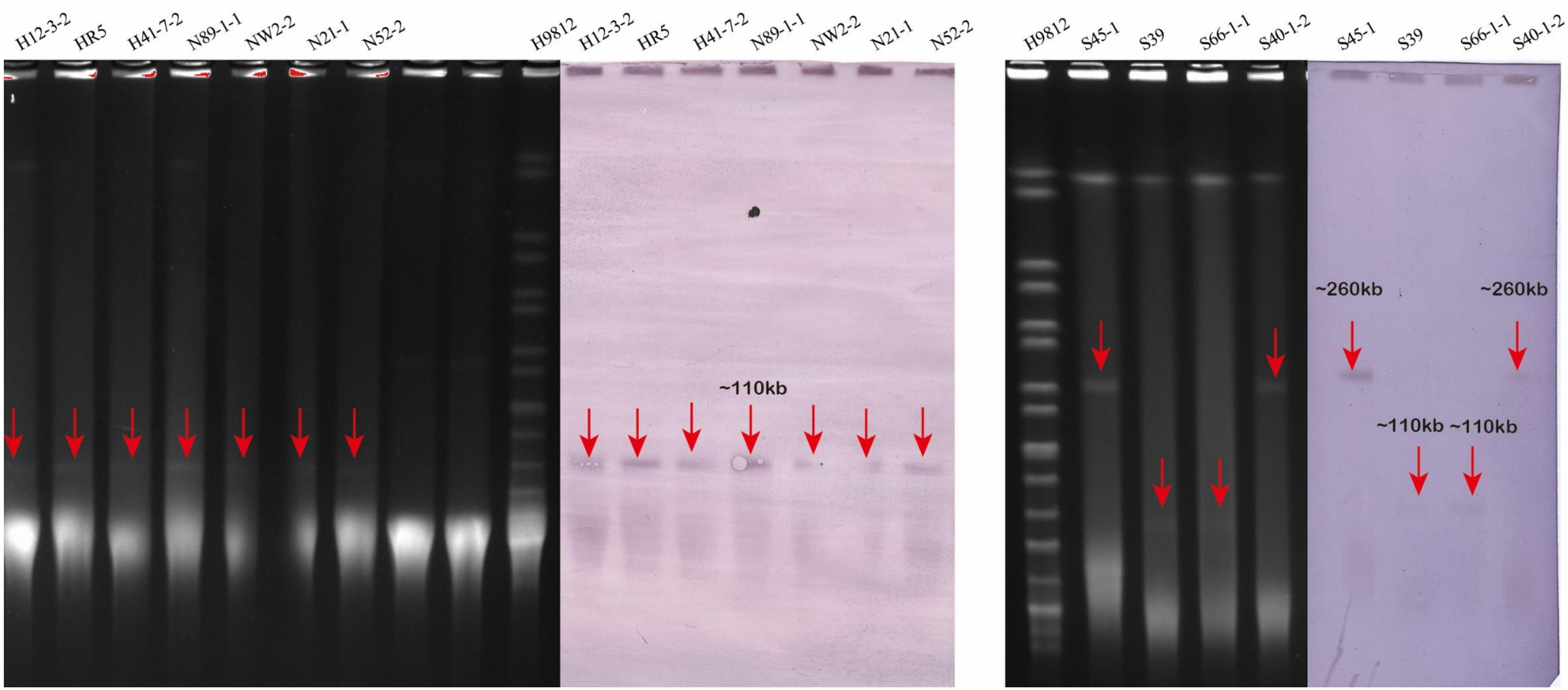

Figure S1. S1-PFGE and Southern blots hybridization profiles of *fosA3*-carrying plasmids in the 11 transformants.
